# Naringenin Attenuates Myocardial Ischemia-Reperfusion Injury via cGMP-PKGI*α* Signaling and In Vivo and In Vitro Studies

**DOI:** 10.1155/2019/7670854

**Published:** 2019-01-08

**Authors:** Li-Ming Yu, Xue Dong, Jian Zhang, Zhi Li, Xiao-Dong Xue, Hong-Jiang Wu, Zhong-Lu Yang, Yang Yang, Hui-Shan Wang

**Affiliations:** ^1^Department of Cardiovascular Surgery, General Hospital of Shenyang Military Area Command, 83 Wenhua Road, Shenyang, Liaoning 110016, China; ^2^Department of Neurosurgery, General Hospital of Shenyang Military Area Command, 83 Wenhua Road, Shenyang, Liaoning 110016, China; ^3^Department of Pharmacy, General Hospital of Shenyang Military Area Command, 83 Wenhua Road, Shenyang, Liaoning 110016, China; ^4^Key Laboratory of Resource Biology and Biotechnology in Western China, Ministry of Education, Faculty of Life Sciences, Northwest University, 229 Taibai North Road, Xi'an, Shaanxi 710069, China

## Abstract

Endoplasmic reticulum (ER) stress and oxidative stress contribute greatly to myocardial ischemia-reperfusion (MI/R) injury. Naringenin, a flavonoid derived from the citrus genus, exerts cardioprotective effects. However, the effects of naringenin on ER stress as well as oxidative stress under MI/R condition and the detailed mechanisms remain poorly defined. This study investigated the protective effect of naringenin on MI/R-injured heart with a focus on cyclic guanosine monophosphate- (cGMP-) dependent protein kinase (PKG) signaling. Sprague-Dawley rats were treated with naringenin (50 mg/kg/d) and subjected to MI/R surgery with or without KT5823 (2 mg/kg, a selective inhibitor of PKG) cotreatment. Cellular experiment was conducted on H9c2 cardiomyoblasts subjected to simulated ischemia-reperfusion treatment. Before the treatment, the cells were incubated with naringenin (80 *μ*mol/L). PKGI*α* siRNA was employed to inhibit PKG signaling. Our in vivo and in vitro data showed that naringenin effectively improved heart function while it attenuated myocardial apoptosis and infarction. Furthermore, pretreatment with naringenin suppressed MI/R-induced oxidative stress as well as ER stress as evidenced by decreased superoxide generation, myocardial MDA level, gp91*^phox^* expression, and phosphorylation of PERK, IRE1*α*, and EIF2*α* as well as reduced ATF6 and CHOP. Importantly, naringenin significantly activated myocardial cGMP-PKGI*α* signaling while inhibition of PKG signaling with KT5823 (in vivo) or siRNA (in vitro) not only abolished these actions but also blunted naringenin's inhibitory effects against oxidative stress and ER stress. In summary, our study demonstrates that naringenin treatment protects against MI/R injury by reducing oxidative stress and ER stress via cGMP-PKGI*α* signaling. Its cardioprotective effect deserves further clinical study.

## 1. Introduction

Acute myocardial infarction (AMI) remains one of the leading causes of death worldwide. Although restoration of blood flow as soon as possible, named reperfusion, has been established as the major strategy to treat AMI, it can result in remarkable cardiac damage [1]. Despite that efforts have been made to explore the rescue approaches for treating myocardial ischemia-reperfusion (MI/R) injury, further studies are still needed to find the effective therapeutic strategies [2, 3].

MI/R injury is a quite intricate process involving numerous mechanisms. It has been found that oxygen free radical accumulation, calcium overload, endoplasmic reticulum (ER) stress, and apoptosis all play a key role in the etiopathogenesis of MI/R injury [4]. Previously, we and others have found that severe ER stress is one of the lethal contributors to cardiomyocyte death under stress conditions [5–8]. During MI/R, robust oxygen free radical generation and the depletion of oxygen/glucose supply cause the production of nitric oxide (NO) and other reactive oxygen species (ROS) [9]. The changes in cellular redox regulation interfere with the function of disulphide bonding in the lumen of the ER, initiating the complex unfolded protein response (UPR). The UPR favors cellular homeostasis; however, prolonged ER stress can trigger cardiomyocyte apoptosis [10, 11]. Thus, pharmacologic inhibition of oxidative damage as well as sustained ER stress has been proved to confer cardioprotection [10, 12–14].

**Figure 1:**
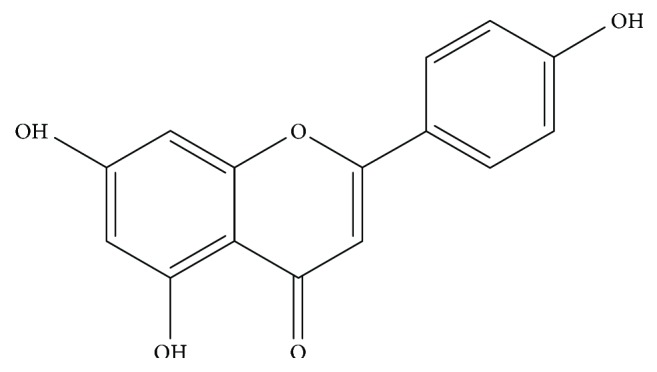
Molecular structure of naringenin (NRG).

Natural flavonoids have been shown to exhibit safe and effective cardioprotective properties [15]. Several recent researches indicated that naringenin (NRG, Figure 1), a flavonoid derived from the Citrus genus, exerted beneficial effects on the cardiovascular system [16, 17]. These effects may be due, at least in part, to its antioxidative properties. For example, one study by Meng et al. demonstrated that naringenin protected against MI/R injury through activating ATP-sensitive potassium channels and enhancing myocardial antioxidant capacity [18]. Intriguingly, in cultured H9c2 cells, Tang and coworkers found that naringenin regulated cellular ER stress level and attenuated hypoxia/reoxygenation-induced cytotoxicity [19]. However, the detailed mechanisms of naringenin's inhibitory effects against oxidative stress injury under MI/R conditions remain uncovered. Furthermore, whether naringenin attenuates ER stress induced by MI/R injury in experimental animal models and the underlying mechanism are also unknown.

Cyclic guanosine monophosphate- (cGMP-) dependent protein kinase (PKG) signaling has been suggested as a therapeutic target for MI/R injury [20]. The cardiovascular beneficial actions of several biological messengers (such as nitric oxide and carbon monoxide) are facilitated through activation of soluble guanylyl cyclase (sGC) with subsequent synthesis of second messenger cGMP [20–24]. As a well-characterized downstream effector of cGMP, PKGI is centrally involved in regulating intracellular on/off switches that help maintain dynamical redox balances [20, 21]. Previously, we demonstrated that exogenous activation of cGMP-PKGI*α* signaling conferred cardioprotection via activating nuclear factor-erythroid-2- (NF-E2-) related factor 2 (NRF-2) as well as modulating mitogen-activated protein kinase (MAPK) pathway [25]. Furthermore, we and others have found that cGMP-PKGI*α* signaling might also regulate cardiomyocyte ER stress level under stress conditions [5, 26–28]. However, whether cGMP-PKGI*α* signaling is involved in the cardioprotective actions of naringenin and the underlying mechanisms have not been previously investigated.

In the present study, we designed in vivo and in vitro studies to (i) investigate the effects of naringenin treatment on myocardial oxidative stress and ER stress damage during MI/R injury and (ii) determine the potential role of the cGMP-PKG signaling pathway as well as the underlying mechanisms during this process.

## 2. Materials and Methods

### 2.1. Reagents and Chemicals

Naringenin (NRG) was acquired from Aladdin Biotechnology (Shanghai, China). KT5823, 3-(4,5-dimethylthiazol-2-yl)-2,5-diphenyltetrazolium bromide (MTT) assay kit, and protease inhibitor cocktail were acquired from Sigma-Aldrich (St. Louis, MO, USA). Terminal deoxynucleotidyl transferase-mediated dUTP nick end labeling (TUNEL) assay kit was obtained from Roche Molecular Biochemicals (Mannheim, Germany). Evans blue and triphenyltetrazolium chloride (TTC) were obtained from Solarbio Technology (Beijing, China). The assay kits for lactate dehydrogenase (LDH), superoxide, and malondialdehyde (MDA) were obtained from Jiancheng Bioengineering Institute (Nanjing, China). The cGMP ELISA kit was obtained from Enzo Life Sciences (Ann Arbor, MI, USA). BCA protein quantification kit was purchased from Thermo Fisher Scientific (Waltham, MA, USA). H9c2 cardiomyoblast cells were acquired from Tiancheng Biotechnology (Shanghai, China). Dulbecco's modified Eagle's medium and penicillin/streptomycin solution were purchased from HyClone (Logan, UT, USA). The transfection reagent and small interfering RNA duplex for PKGI*α* were purchased from Santa Cruz Biotechnology (CA, USA). The primary antibodies against caspase-3, cleaved caspase-3, protein kinase RNA-like endoplasmic reticulum kinase (PERK), p-PERK (Thr980), eukaryotic translation initiation factor 2 subunit-*α* (EIF2*α*), p-EIF2*α* (Ser51), VASP, and p-VASP (Ser239) were purchased from Cell Signaling Technology (Danvers, MA, USA). The primary antibodies against inositol-requiring protein-1*α* (IRE1*α*) and p-IRE1*α* (Ser724) were purchased from Abcam biotechnology (Abcam, USA). The other primary antibodies were all obtained from Santa Cruz Biotechnology (CA, USA). Rabbit anti-goat, goat anti-rabbit, and goat anti-mouse secondary antibodies were acquired from Zhongshan Company (Beijing, China).

### 2.2. Animals

This study was approved by the Animal Ethics Committee of General Hospital of Shenyang Military Area Command and carried out in adherence with the Guide for the Care and Use of Laboratory Animals issued by the US National Institutes of Health (NIH Publication, 8th Edition, 2011). Male Sprague-Dawley (SD) rats weighing 200 g to 220 g were purchased from the Experimental Animal Center of the General Hospital of Shenyang Military Area Command. The animals were maintained at 22-24 degree Celsius under a cycle of 12 h: 12 h light-dark with free access to food and water [25].

### 2.3. Myocardial Ischemia-Reperfusion Surgery and Experimental Setup

All surgical procedures were performed under sterile conditions as described in our previous research [7, 29, 30]. The experiment was designed according to recent guidelines [31]. In brief, SD rats were anesthetized with sodium pentobarbital (40 mg/kg, i.p.) and artificially ventilated. The three-lead electrocardiogram was employed to monitor the heartbeat as well as the typical ECG changes at the beginning of myocardial ischemia. A microcatheter (Taimeng Technology, Chengdu, China) was inserted into the left ventricle through the right carotid artery to evaluate cardiac function during the surgery. After these treatments, MI/R surgery was performed. Electrocardiogram and ventricular functional parameters were continuously monitored using the hemodynamic monitoring system (PowerLab, ADInstruments technology, Castle Hill, Australia). Myocardial ischemia was induced by ligation of the left anterior descending coronary artery with a slipknot as described in our previous work [30]. After 30 minutes of ischemia, the slipknot was released. The myocardium was reperfused for 4 h (for cell apoptosis, protein analysis, and cardiac function measurements) or 6 h (for infarct size measurement). The animals in the sham group underwent the same surgical procedures except that the suture placed under the left anterior descending coronary artery was not tied. Before the surgery, naringenin (50 mg/kg/d, diluted in sterile water) or vehicle (1.5 mL, sterile water) was administered by oral gavage for 5 days. KT5823 (2 mg/kg, a selective inhibitor of PKG) was intravenously administered to the animals at the beginning of myocardial ischemia. The dosage of exogenous agents was chosen based on preliminary experimental results and our previous work [5, 25, 32, 33].

### 2.4. Cell Culture and Simulated Ischemia-Reperfusion Treatment

The H9c2 cell line, a subclone of the original clonal cell line derived from embryonic rat heart tissue, was widely used to study myocardial cell ischemia. In the present study, the cells were cultured in Dulbecco's modified Eagle's medium (DMEM) supplemented with 10% fetal bovine serum (FBS), 100 U/mL of penicillin, and 100 U/mL of streptomycin as previously described [25]. The culturing system was maintained in a humidified atmosphere with 5% CO_2_ at 37°C. Simulated ischemia-reperfusion (SIR) treatment was carried out using physiological concentrations of potassium, lactate, and hydrogen as described in our previous work [29]. In brief, the cells were exposed to an ischemic buffer containing (in mmol/L) 137 NaCl, 12 KCl, 0.49 MgCl_2_, 0.9 CaCl_2_, 4 HEPES, 10 deoxyglucose, 0.75 sodium dithionate, and 20 lactate (pH 6.5) for 1 hour in a humidified cell culture incubator (21% O_2_, 5% CO_2_, 37°C). Then, the medium was replaced with normal serum-free DMEM for 4 hours to simulate reperfusion treatment. Finally, the cells were washed with phosphate-buffered saline (PBS) and prepared for further analysis. Before the simulated ischemia, the cells were incubated in serum-free DMEM [supplemented with vehicle (0.1% DMSO) or naringenin (80 *μ*mol/L)] for 6 h.

### 2.5. Myocardial Apoptosis and Infarction Determination

The TUNEL assay was performed in a blinded fashion to determine myocardial apoptosis as described previously [12]. The TUNEL-positive cells were counted under a high-power field (magnification ×200). Apoptotic index was calculated automatically as the ratio of TUNEL-positive nuclei/total number of nuclei × 100%. The Evans Blue/TTC double-staining technique was used to determine myocardial infarct size (INF) as described in our previous work [8]. After 6 hours of reperfusion, the suture under the left anterior descending coronary artery was retied and 1 mL 2% Evans Blue dye was injected into the aorta. The dye was uniformly distributed in the myocardium except in the portion of the heart that was previously perfused by the occluded coronary artery (area-at-risk (AAR)). Then, the heart was sliced into 1 mm thick sections by using a heart slice chamber. Slices were incubated in 1% TTC in phosphate buffer (pH 7.4) for 15 min and photographed with a digital camera. The Evan's Blue-stained area (area-not-at-risk (ANAR)), TTC-stained area (ischemic but viable tissue), and TTC staining-negative area (infarct myocardium) were measured digitally using Image-Pro Plus software (Media Cybernetics). The myocardial infarct size was expressed as a percentage of infarct area (INF) over total AAR (INF/AAR × 100%).

### 2.6. Myocardial Superoxide Generation and Malondialdehyde Content Measurement

Superoxide generation in tissue or cells was measured by lucigenin-enhanced chemiluminescence as described previously [8]. Superoxide generation was expressed as relative light units (RLU) per second per milligram heart weight (RLU/mg/s). The malondialdehyde (MDA) content measurement was determined spectrophotometrically following the manufacture's instruction as previously described [12].

### 2.7. cGMP Measurement

Cyclic GMP in tissue or cells was determined using a commercially available cGMP complete ELISA kit following the manufacturer's instructions as described previously [25]. Results were presented as pmol cGMP/mg protein.

### 2.8. Cell Viability Assessment

MTT assay was carried out to measure H9c2 viability as described in our previous work [7, 25]. The cell viability was calculated by dividing the optical density of samples with the optical density of the control group.

### 2.9. Small Interfering RNA Transfection

The siRNA transfection solutions (including PKGI*α* siRNA duplex, control siRNA duplex, siRNA transfection medium, and siRNA transfection reagent) was purchased from Santa Cruz Biotechnology (CA, USA) and carried out as described in our previous work [34]. The cells were incubated for additional 18 hours and subsequently prepared for use in further experiments. The knockdown capacity of PKGI*α* siRNA was assessed by Western blot analysis.

### 2.10. Western Blot Analysis

The expressions of caspase-3, cleaved caspase-3, PERK, p-PERK (Thr980), IRE1*α*, p-IRE1*α* (Ser724), EIF2*α*, p-EIF2*α* (Ser51), CCAAT/enhancer-binding protein homologous protein (CHOP), activating transcription factor 6 (ATF6), gp91*^phox^*, catalase, MnSOD, PKGI*α*, vasodilator-stimulated phosphoprotein (VASP), p-VASP (Ser239), and *β*-actin were measured using Western blot as described previously [34]. The protein samples were separated by electrophoresis on SDS-PAGE and transferred to a polyvinylidene difluoride membrane. The membranes were blocked with 5% milk and then incubated overnight with the appropriate primary antibodies. Then, they were washed and incubated with the corresponding HRP-conjugated secondary antibodies. The blots were visualized with ECL Plus reagent and quantified using an image analyzer (Tanon Technology, Shanghai, China). *β*-Actin was used as internal loading control. The value for the sham or control group was defined as 1.

### 2.11. Western Blot Stripping and Reprobing Protocol

The classic stripping buffer (SB, 100 mL) for Western blot membranes were prepared by mixing 6.25 mL of 1 M Tris-HCl (pH 6.8), 10 mL of 20% SDS, and 700 *μ*L *β*-mercaptoethanol together. Then, the mixture was diluted to 100 ml with deionized water. After the membranes were exposed, they were washed and incubated in fresh-prepared SB for 45 min at 50°C (with slight agitation). Then, the membranes were rinsed under running water for 1 h and 5 times for 5 min with Tris-buffered saline with Tween 20 (TBST) buffer. Finally, they were blocked with 5% bovine serum albumin (BSA), incubated with target primary antibodies as well as corresponding secondary antibodies, and then reprobed.

### 2.12. Statistical Analysis

All results are presented as mean ± SEM. Data were subjected to ANOVA followed by Bonferroni correction for post hoc *t* test. *P* values <0.05 were considered statistically significant.

## 3. Results

### 3.1. Effect of Naringenin and KT5823 on Cardiac Function, Myocardial Apoptosis, and Myocardial Infarction

**Figure 2:**
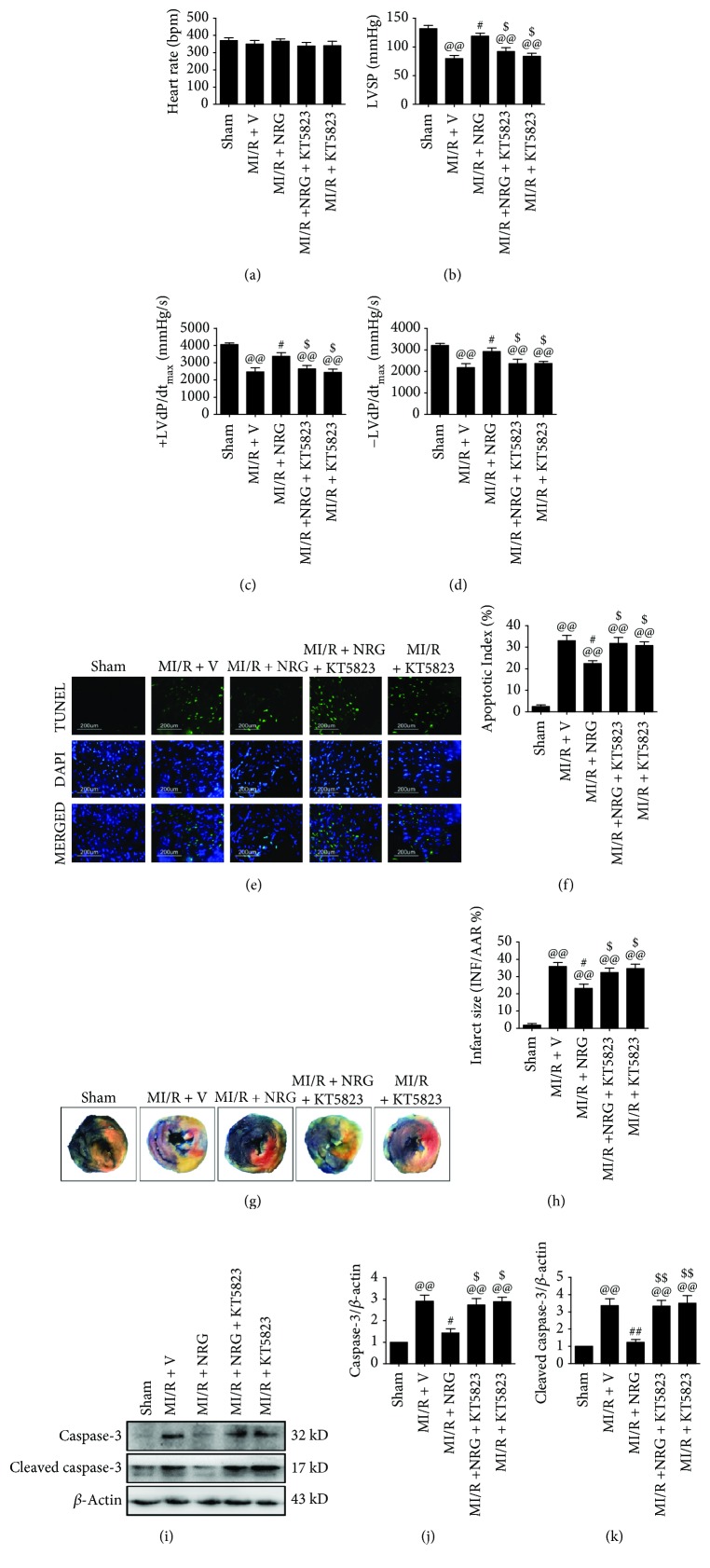
Evaluation of cardiac function, myocardial apoptosis, and infarction. The animals were given naringenin treatment by oral gavage at a dose of 50 mg/kg/d for 5 days and then subjected to MI/R surgery. KT5823 (2 mg/kg) was intravenously administered to the animals at the beginning of myocardial ischemia. Cardiac function and myocardial apoptosis were measured after 4 hours of reperfusion. Myocardial infarct size was measured after 6 hours of reperfusion. (a) Heart rate (HR). (b) Left ventricular systolic pressure (LVSP). (c, d) The instantaneous first derivative of left ventricular pressure (+d*P*/dt_max_ and −d*P*/dt_max_). (e) Representative photomicrographs of TUNEL staining (200x). Green fluorescence shows TUNEL-positive nuclei; blue fluorescence shows nuclei of total cardiomyocytes. (f) Percentage of TUNEL-positive nuclei. (g) Representative photographs of Evan's Blue-TTC staining. (h) Myocardial infarct size expressed as percentage of area-at-risk (AAR). (i) Representative blots. (j) Caspase-3 expression. (k) Cleaved caspase-3 expression. Data are expressed as mean ± SEM. *n* = 6 per group. ^@@^*P* < 0.01*vs.* sham group, ^#^*P* < 0.05/^##^*P* < 0.01*vs.* MI/R + V group, and ^$^*P* < 0.05/^$$^*P* < 0.01*vs.* MI/R + NRG group. MI/R: myocardial ischemia-reperfusion; V: vehicle; NRG: naringenin.

To examine the influence of naringenin and PKG signaling inhibitor on cardiac damage after MI/R surgery, we treated the rats with naringenin at a dose of 50 mg/kg/d for 5 days and then performed MI/R surgery on them in the presence or absence of KT5823 (2 mg/kg). As seen in Figure 2(a), neither naringenin nor KT5823 significantly affected the heart rate (compared with the MI/R + V group). However, naringenin treatment improved cardiac function and reduced myocardial infarction after ischemia-reperfusion insult, as evidenced by increased left ventricular systolic pressure (LVSP), instantaneous first derivative of left ventricular pressure (+d*P*/dt_max_ and −d*P*/dt_max_), as well as decreased infarct size (Figures 2(b)-2(d), 2(g), and 2(h), compared with the MI/R + V group). Additionally, naringenin also exhibited a markedly antiapoptotic effect by inhibiting myocardial apoptosis and suppressing cleaved caspase-3 as well as caspase-3 expressions (Figures 2(e), 2(f), and 2(i)-2(k), compared with the MI/R + V group). Although KT5823 alone did not cause significant cardiac damage (compared with the MI/R + V group), KT5823 markedly inhibited naringenin's cardioprotective actions (compared with the MI/R + NRG group). These results indicated that PKG signaling might play a key role during this process.

### 3.2. Effect of Naringenin and KT5823 on Myocardial ER Stress

**Figure 3:**
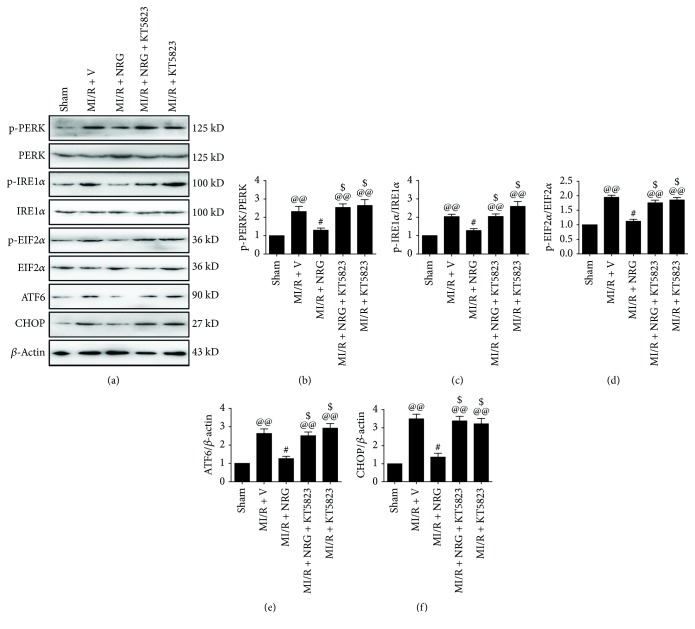
Evaluation of myocardial endoplasmic reticulum stress level. Western blot analysis was conducted after 4 hours of reperfusion. (a) Representative blots, (b) PERK (Thr980) phosphorylation level, (c) IRE1*α* (Ser724) phosphorylation level, (d) EIF2*α* (Ser51) phosphorylation level, (e) ATF6 expression, and (f) CHOP expression. Data are expressed as mean ± SEM. *n* = 6 per group. ^@@^*P* < 0.01*vs.* sham group, ^#^*P* < 0.05/^##^*P* < 0.01*vs.* MI/R + V group, and^$^*P* < 0.05/^$$^*P* < 0.01*vs.* MI/R + NRG group. MI/R: myocardial ischemia-reperfusion; V: vehicle; NRG: naringenin.

Previously, we and others have demonstrated that ER stress contributed to cardiomyocyte death during MI/R injury [5, 6, 12]. MI/R injury is known to result in persistent unfolded protein response (UPR) and eventually leads to cellular apoptosis [10, 11]. Thus, we focused on ER stress-related protein expression in the present study. Enhanced myocardial ER stress was found in the MI/R-injured group (Figure 3). The phosphorylation levels of PERK, IRE1*α*, and EIF2*α* were significantly increased in the MI/R + V group (compared with the sham group). Meanwhile, ATF6 and CHOP were also upregulated in the MI/R + V group (compared with the sham group). Interestingly, we found that naringenin significantly suppressed myocardial ER stress as evidenced by reduced PERK, IRE1*α*, and EIF2*α* phosphorylation as well as decreased ATF6 and CHOP protein levels (compared with the MI/R + V group). However, these effects were abolished by KT5823 administration, suggesting that PKG signaling played a role in mediating naringenin's inhibitory effect against myocardial ER stress.

### 3.3. Effect of Naringenin and KT5823 on Cardiac Oxidative Stress Damage and cGMP-PKGI*α* Pathway

**Figure 4:**
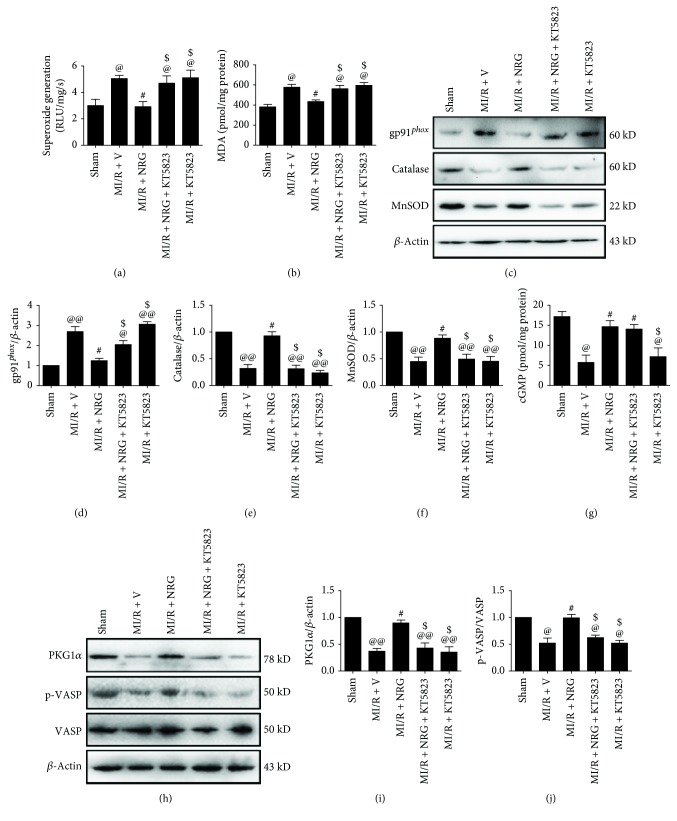
Evaluation of myocardial oxidative stress markers and cGMP-PKGI*α* signaling. All the measurements were carried out after 4 hours of reperfusion. (a) Myocardial superoxide generation, (b) myocardial malondialdehyde (MDA) contents, (c) representative blots, (d) gp91*^phox^* expression, (e) catalase expression, (f) MnSOD expression, (g) myocardial cGMP level, (h) representative blots, (i) PKGI*α* expression, and (j) VASP (Ser239) phosphorylation. Data are expressed as mean ± SEM*n* = 6 per group. ^@^*P* < 0.05/^@@^*P* < 0.01*vs.* sham group, ^#^*P* < 0.05*vs.* MI/R + V group, and ^$^*P* < 0.05/^$$^*P* < 0.01*vs.* MI/R + NRG group. MI/R: myocardial ischemia-reperfusion; V: vehicle; NRG: naringenin.

To further evaluate the potential effect of naringenin on MI/R injury, we evaluated cardiac oxidative stress damage in this setting. As seen in Figures 4(a) and 4(b), MI/R injury significantly aggravated oxidative stress by increasing superoxide generation and myocardial MDA level. The expression of gp91*^phox^* (a key component of NADPH oxidase which is the most important superoxide-producing enzyme in the ischemic reperfused heart) was also markedly upregulated while catalase and MnSOD levels were downregulated in the MI/R-treated group (Figures 4(c)-4(f), compared with the sham group). Naringenin exhibited an effective antioxidative effect by reducing superoxide as well as MDA content and reversing the expression of gp91*^phox^*, catalase, and MnSOD (compared with the MI/R + V group). However, KT5823 also significantly inhibited these actions.

Next, we explored the underlying mechanisms with a focus on the myocardial cGMP-PKGI*α* pathway. As shown in Figures 4(g)-4(j), MI/R insult markedly reduced cGMP-PKGI*α* signaling as evidenced by decreased myocardial cGMP level and downregulated PKGI*α* expression and VASP phosphorylation at Ser239 (a key marker for evaluating PKG activation). However, naringenin treatment effectively activated cGMP-PKGI*α* signaling by increasing cGMP as well as PKGI*α* and the p-VASP/VASP ratio (compared with the MI/R + V group), indicating that cGMP-PKGI*α* might contribute to the myocardial protective actions of naringenin. As expected, although KT5823 caused no significant changes in myocardial cGMP level, it markedly reduced PKGI*α* expression as well as its activity (compared with the MI/R + NRG group).

### 3.4. Effect of Naringenin and PKGI*α* siRNA on Cellular Damage after Simulated Ischemia-Reperfusion Insult

**Figure 5:**
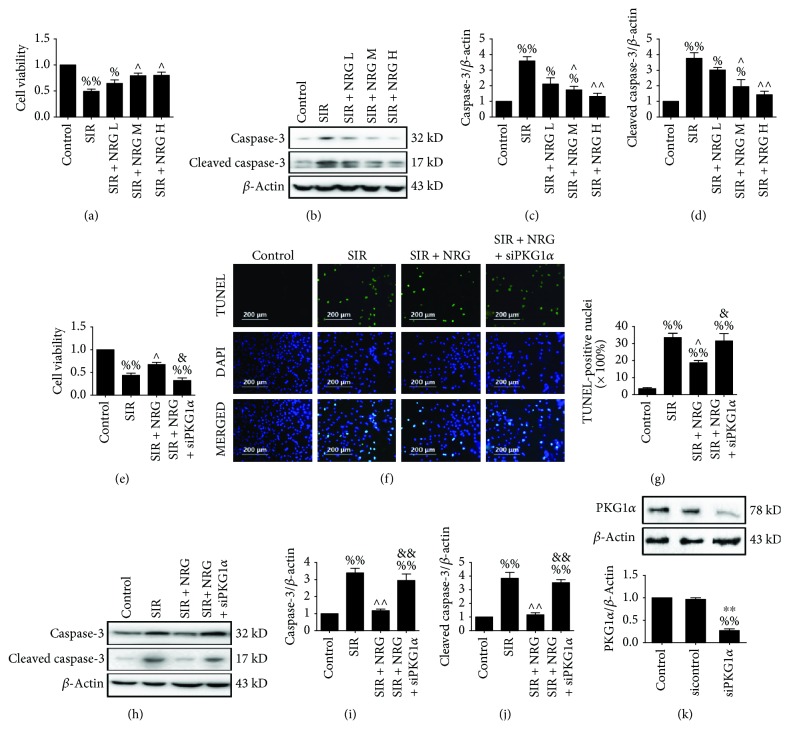
Evaluation of cell viability and apoptosis. Initially, H9c2 cardiomyoblasts received low (L), medium (M), or high (H) concentrations of naringenin treatment (40, 80, or 160 *μ*mol/L) for 6 hours and subjected to simulated ischemia-reperfusion injury. Cell viability and apoptosis were assessed (a-d). Next, H9c2 cells were transfected with PKGI*α* siRNA and administered with or without naringenin (80 *μ*mol/L, 6 hours). Then, they emulated ischemia-reperfusion SIR treatment. Cell viability and apoptosis were measured after 4 hours of simulated reperfusion (e-j). (a) Cell viability, (b) representative blots, (c) caspase-3 expression, (d) cleaved caspase-3 expression, (e) cell viability, and (f) representative photomicrographs of TUNEL staining (200x). Green fluorescence shows TUNEL-positive nuclei; blue fluorescence shows nuclei of total cardiomyocytes. (g) Percentage of TUNEL-positive nuclei, (h) representative blots, (i) caspase-3 expression, (j) cleaved caspase-3 expression, (k) the knockdown capacity of PKGI*α* siRNA were evaluated by Western blotting. Data are expressed as mean ± SEM. *n* = 6 per group. ^%^*P* < 0.05/^%%^*P* < 0.01*vs.* control group, ^∧^*P* < 0.05/^∧∧^*P* < 0.01*vs.* SIR group, ^&^*P* < 0.05/^&&^*P* < 0.01*vs.* SIR + NRG group, and ^∗∗^*P* < 0.01*vs.* sicontrol group. SIR: simulated myocardial ischemia-reperfusion; NRG: naringenin.

To further assess the effect of naringenin on cardiac damage during the ischemia-reperfusion period, we conducted an in vitro experiment using H9c2 cells. As shown in Figures 5(a)-5(d), we found that naringenin showed a dose-dependent attenuation of SIR-induced cell viability reduction and apoptosis with a maximum protection at 160 *μ*mol/L. In fact, treatment with 80 *μ*mol/L of naringenin significantly increased cell viability while it reduced the apoptotic index as well as caspase-3 and cleaved caspase-3 expressions (Figures 5(e)-5(j)). In the control group, we found that PKGI*α* small interfering RNA transfection markedly suppressed PKGI*α* expression while control small interfering RNA had no significant effect (Figure 5(k), compared with the control group). As expected, the improved cell viability was remarkably reduced by PKGI*α* siRNA treatment (Figure 5(e), compared with the SIR + NRG group). Consistent with the in vivo results, PKGI*α* knockdown also induced aggravated cellular apoptosis as evidenced by increased percentage of TUNEL-positive nuclei and upregulated caspase-3 as well as cleaved caspase-3 levels (Figures 5(f)-5(j), compared with the SIR + NRG group).

### 3.5. Effect of Naringenin and PKGI*α* siRNA on Cellular ER Stress after Simulated Ischemia-Reperfusion Insult

**Figure 6:**
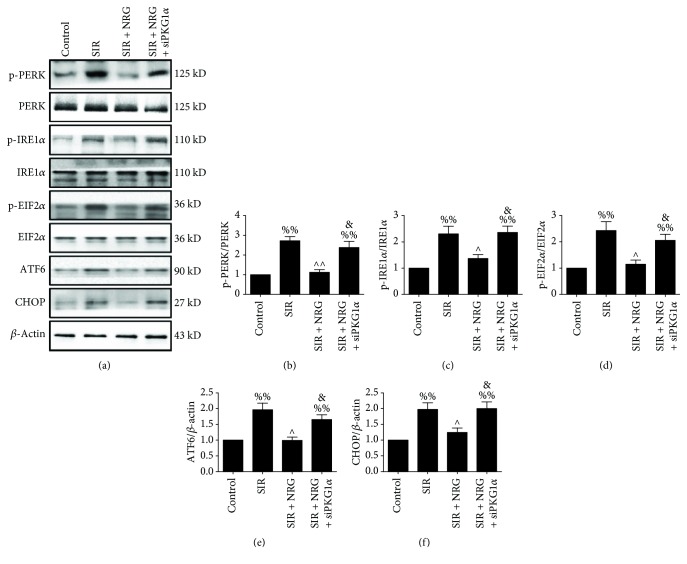
Evaluation of cellular endoplasmic reticulum stress level. All the measurements were carried out after 4 hours of simulated reperfusion. (a) Representative blots, (b) PERK (Thr980) phosphorylation level, (c) IRE1*α* (Ser724) phosphorylation level, (d) EIF2*α* (Ser51) phosphorylation level, (e) ATF6 expression, and (f) CHOP expression. Data are expressed as mean ± SEM. *n* = 6 per group. ^%%^*P* < 0.01*vs.* control group, ^∧^*P* < 0.05/^∧∧^*P* < 0.01*vs.* SIR group, and ^&^*P* < 0.05*vs.* SIR + NRG group. SIR: simulated myocardial ischemia-reperfusion; NRG: naringenin.

Next, we evaluated ER stress-related protein expressions in cultured cells subjected to SIR injury. As shown in Figures 6(a)-6(d), we found a remarkable reduction in the phosphorylation levels of PERK, IRE1*α*, and EIF2*α* in the naringenin-treated group (compared with the SIR group). These effects were reversed by PKGI*α* knockdown. Meanwhile, PKGI*α* siRNA transfection also blunted the suppressive effect of naringenin on ATF6 and CHOP expressions (Figures 6(e) and 6(f), compared with the SIR + NRG group). These results further confirmed that the ameliorative effect on ER stress contributed to the cellular protective of naringenin against SIR damage. Importantly, PKGI*α* signaling played a key role in this setting.

### 3.6. Effect of Naringenin and PKGI*α* siRNA on Cellular Oxidative Stress and cGMP-PKG Signaling Pathway after Simulated Ischemia-Reperfusion Insult

**Figure 7:**
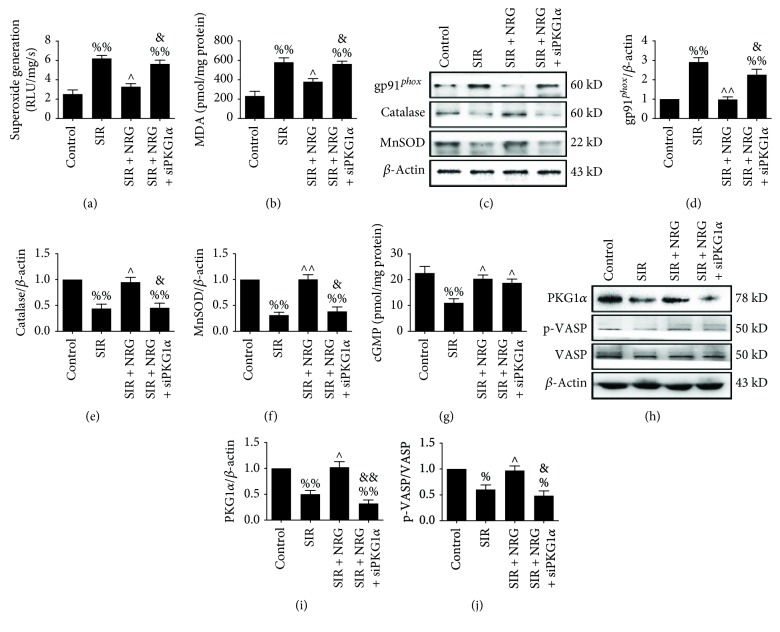
Evaluation of myocardial oxidative stress markers and cGMP-PKGI*α* signaling. All the measurements were carried out after 4 hours of simulated reperfusion. (a) Cellular superoxide generation, (b) cellular malondialdehyde (MDA) contents, (c) representative blots, (d) gp91*^phox^* expression, (e) catalase expression, (f) MnSOD expression, (g) cellular cGMP level, (h) representative blots, (i) PKGI*α* expression, and (j) VASP (Ser239) phosphorylation. Data are expressed as mean ± SEM. *n* = 6 per group. ^%^*P* < 0.05/^%%^*P* < 0.01 *vs.* control group, ^∧^*P* < 0.05/^∧∧^*P* < 0.01*vs.* SIR group, and ^&^*P* < 0.05/^&&^*P* < 0.01*vs.* SIR + NRG group. SIR: simulated myocardial ischemia-reperfusion; NRG: naringenin.

To determine the detailed mechanisms, we measured oxidative stress level in cultured cells. As shown in Figures 7(a) and 7(b), naringenin significantly decreased cellular superoxide and MDA content (compared with the SIR group). The expression of gp91*^phox^* was also reduced while the protein levels of catalase as well as MnSOD were enhanced in the naringenin-treated group (Figures 7(c)-7(f), compared with the SIR group). These data confirmed that naringenin exerted a profound antioxidative effect during ischemia-reperfusion injury. However, the protective effects were abolished by PKGI*α* siRNA transfection, suggesting that PKGI*α* signaling played a critical role in mediating naringenin's antioxidant actions. Consistent with the in vivo results, the marked upregulation of cellular cGMP level, PKGI*α* expression, and VASP phosphorylation at Ser239 was found in the naringenin-treated group (Figures 7(g)-7(j), compared with the SIR + NRG group). Although PKGI*α* siRNA had no significant effect on cGMP level, it effectively suppressed PKGI*α* expression and the p-VASP/VASP ratio (Figures 7(g)-7(j), compared with the SIR + NRG group). Taken together, these results demonstrated that naringenin exerted a profound ameliorative effect against oxidative stress and endoplasmic reticulum stress-induced by myocardial ischemia-reperfusion injury via the cGMP-PKG signaling pathway.

### 3.7. Discussion

The results of the present study revealed that naringenin could ameliorate MI/R-induced myocardial injury through activating cGMP-PKG signaling. Additionally, the cGMP-PKG pathway played a critical role in mediating the suppressive effects of naringenin against oxidative stress and ER stress damage during this process.

Naringenin (4′,5,7-thrihydroxyflavanone), is a citrus flavanone derived in fruits and vegetables like oranges, grapefruit, and lemons [35]. As one of the most consumed flavonoids in the society with good bioavailability [17, 36], naringenin has attracted more and more attention for its disease-preventing properties, such as anti-inflammatory [37, 38], antioxidative [38–40], anticancer [41], and, importantly, cardioprotective properties [18, 33, 42]. Previously, naringenin has been postulated as a potential therapeutic agent in oxidative stress-related conditions. For example, naringenin was demonstrated to enhance learning acquisition and memory retention by improving antioxidant enzyme activities and increasing antioxidant compound concentration in rats [43]. Roy et al. also found that naringenin treatment attenuated streptozotocin-induced diabetic rat renal impairment by suppressing oxidative stress damage [44]. In this study, we found that during MI/R injury, the naringenin-treated group exhibited significantly enhanced antioxidant enzyme expressions and reduced myocardial oxidative stress level. Such beneficial effects of naringenin regarding antioxidant activity under myocardial stress conditions were reported in previous researches [32, 39, 40, 45, 46]. These studies indicated that naringenin exerted profound antioxidative effects and improved cardiac functional recovery after MI/R injury. These data all showed that naringenin, through its antioxidant effects, may represent a novel therapeutic option to protect against ischemic heart disease.

Multiple events participate in MI/R injury. Most, if not all, of these processes are potent inducers of the unfolded protein response, a cellular mechanism evolved to cope with protein-folding stress [47, 48]. Under physiological conditions, ER serves as an important membranous organelle with an essential role in multiple cellular activities including nascent polypeptide folding, assembly, modification and secretion, lipid synthesis, and calcium storage. However, when ER is exposed to stress stimuli, such as ischemia-reperfusion, oxygen free radical exposure, and disturbance of calcium balance, the homeostasis of it is damaged which further results in the accumulation of unfolded/misfolded proteins. These changes can potentially induce ER dysfunction, collectively known as ER stress. It is noted that moderate ER stress can be detected by the transmembrane protein sensors (PERK, IRE1, and ATF6) of ER and initiates the UPR to recover the ER homeostasis [11]. However, if the stress persists, UPR can trigger the cellular apoptotic pathway and eventually causes cell death. Considerable evidence demonstrated that during the late phase of MI/R, ER stress plays a pivotal role in cardiomyocyte death. In the present study, we showed that after 4 hours of myocardial reperfusion, the phosphorylation level of PERK and IRE1*α* and the expression of ATF6 were significantly increased. As the critical downstream target of PERK, EIF2*α* can be phosphorylated by PERK and promotes prosurvival (early) and proapoptotic (late) transcriptional programs during UPR [49]. Meanwhile, as the common element following activation of the three transmembrane protein sensors, CHOP acts as the critical mediator of cellular apoptosis following ER stress [49]. In this study, we found that EIF2*α* and CHOP were also activated in the MI/R group. Intriguingly, these changes were reversed by naringenin treatment, indicating that naringenin exerted profound inhibitory effects against ER stress under MI/R condition. Although Tang et al. found that naringenin reduced ER stress in a hypoxia/reoxygenation-injured H9c2 cell model [19], our study is the first to assess the modulatory effects of naringenin on MI/R-induced ER stress in an animal model. Notably, Karuppagounder et al. previously reported that naringenin suppressed daunorubicin-induced nephrotoxicity by reducing ER stress level [50]. Another research by Lin et al. showed that naringenin inhibits alcoholic injury by improving lipid metabolism and reducing ER stress in zebrafish larvae [51]. Combined with our in vivo and in vitro finding, all these results implied that naringenin could serve as a novel therapeutic agent against ER stress in the treatment of ischemic heart disease.

Several therapeutic strategies including ischemic preconditioning, nitric oxide, and brain natriuretic peptide (BNP) have been demonstrated to suppress ischemia-reperfusion injury through activation of cGMP-PKG signaling [21, 52–55]. It has been well established that PKGI*α* and PKGI*β* are two major mediators of cGMP signaling. Importantly, PKGI*α* is mainly found in the heart, lung, and cerebellum while PKGI*β* is highly expressed with PKGI*α* in smooth muscle (including uterus, intestine, and trachea) [52]. In fact, cGMP-PKGI*α* signaling has been found to play a critical role during MI/R injury. MI/R could significantly affect the basal myocardial content of cGMP and the activity of PKGI*α*. In the isolated heart, myocardial cGMP level increases during the first 10-25 min of ischemia and decreases thereafter [20, 56]. After 4 hours of reperfusion, markedly reduced myocardial cGMP content and PKGI*α* activity were found in our previous studies [5, 25]. In adult cardiomyocyte, PKGI*α* overexpression protected against SIR-induced cell death [57]. Additionally, PKGI*α* overexpression could initiate multiple intracellular events including activation of nuclear factor erythroid-2-related factor 2 (NRF-2) signaling, opening of mitochondrial K_ATP_ channels, and phosphorylation of AKT and extracellular regulated kinase (ERK), leading to the reduction in oxidative stress damage and cardiomyocyte apoptosis [20, 25, 58–60]. Recently, we also found that the cGMP-PKGI*α* pathway mediated the antioxidative effects of melatonin, the major secretory product synthesized and secreted by the pineal gland, against MI/R injury [25]. In the present study, our in vivo and in vitro data showed that naringenin not only increased myocardial cGMP content but also upregulated PKGI*α* expression as well as the phosphorylation level of VASP (at Ser239), a marker of PKG activity [25, 61]. However, inhibition of PKG signaling with KT5823 (in vivo) or small interfering RNA (in vitro) not only abolished these actions but also blunted naringenin's inhibitory effects on oxidative stress as well as its stimulatory effects on cellular antioxidant enzymes. These findings suggested that naringenin suppressed MI/R-induced oxidative stress damage in a cGMP-PKGI*α*-dependent manner. Although this modulatory effect is predictable since several reports have documented that naringenin might regulate cGMP or PKG signaling in the vascular system [62] and nervous system [63, 64], to the best of our knowledge, the present study is still the first one to elucidate the relationship between naringenin and cGMP-PKGI*α* signaling during ischemic heart disease.

Previously, we and others have found that cGMP-PKG signaling affected myocardial ER function and reduced ER stress level under stress conditions [5, 65]. Gong et al. found that in rats with heart failure, cGMP-specific phosphodiesterase 5 (PDE5, the major enzyme responsible for cGMP hydrolysis) inhibition attenuated ER stress in a PKG-dependent manner [26]. Meanwhile, in a diabetic animal model, we demonstrated that vasonatrin peptide (VNP), the artificial synthetic chimera of atrial natriuretic peptide and C-type natriuretic peptide attenuated MI/R injury by reducing ER stress through cGMP-PKGI*α* signaling [5]. This effect might, at least in part, result from the direct inhibitory actions of oxidative stress damage by cGMP-PKGI*α* activation since enhanced oxidative stress contributed greatly to ER dysfunction during MI/R condition [10, 66, 67]. Intriguingly, in the present study, we demonstrated that cGMP-PKGI*α* signaling also played a central role in mediating the suppressive effect of naringenin on myocardial ER stress during MI/R injury. Inhibition of PKG signaling markedly reversed the suppressive effects of naringenin on the transmembrane protein sensors (PERK, IRE1, and ATF6) of ER as well as myocardial apoptosis.

To date, there were few studies exploring the efficacy of naringenin in the treatment of patients with ischemic heart disease. One study by Piccirillo et al. explored the effects of pink grapefruit juice (which is a recommended dietary addition that contains high amounts of naringenin) on QT variability in patients with dilated or hypertensive cardiomyopathy [68]. Intake of pink grapefruit juice was found to prolong cardiac repolarization and increase temporal cardiac repolarization dispersion. They drew the conclusion that pink grapefruit juice might exert potential proarrhythmic effects in patients with major myocardial structural disorders. This study indicated that, although plenty of basic studies have revealed the beneficial effects of naringenin on cardiovascular performance, further clinical studies are still needed to determine its utility on patients with ischemic heart disease. On the other hand, it has been well established that comorbidities and comedications may disrupt multiple cytoprotective signalings including the cGMP-PKG pathway [69, 70]. Indeed, Giricz et al. demonstrated that hyperlipidaemia induced by a high-cholesterol diet resulted in the deterioration of cGMP-PKG-dependent cardioprotection in rats [71]. Previously, we also showed that diabetic animals exhibited decreased cGMP-PKG signaling activity, while exogenous activation of cGMP-PKG signaling in this circumstance reduced MI/R injury [5]. These data all indicated that cGMP-PKG signaling contributed greatly to the cardioprotective mechanisms under diabetic conditions. However, there are few studies investigating the potential effect of naringenin on cardiovascular complications under the condition of metabolic syndrome. This aspect is also a very important and urgent question for further study.

## 4. Conclusions

Taken together, we demonstrated that naringenin treatment protects against MI/R injury by inhibiting oxidative stress and ER stress via activation of cGMP-PKGI*α* signaling. We think these findings may provide new mechanistic insight into the cardioprotective effects of naringenin, highlighting the opportunity of a novel therapeutic strategy for the patients with ischemic heart disease.

## Data Availability

The data used to support the findings of this study are available from the corresponding author upon request.
